# Co-occurrence of Down Syndrome and Multiple Sclerosis

**DOI:** 10.14789/ejmj.JMJ24-0042-R

**Published:** 2025-03-12

**Authors:** YASUHIRO ARAI, CHIHIRO MANO

**Affiliations:** 1 Division of Child Neurology, Tokyo Metropolitan Tobu Medical Center for Persons with Developmental/Multiple Disabilities, Tokyo, Japan; 1 Division of Child Neurology, Tokyo Metropolitan Tobu Medical Center for Persons with Developmental/Multiple Disabilities, Tokyo, Japan; 2 Department of Pediatric Medicine, Juntendo University Faculty of Medicine, Tokyo, Japan; 2 Department of Pediatric Medicine, Juntendo University Faculty of Medicine, Tokyo, Japan

**Keywords:** Down syndrome, multiple sclerosis, gene dosage effect

## Abstract

**Object:**

Down syndrome (DS) is often associated with autoimmune diseases; however, its association with multiple sclerosis (MS) has rarely been reported. In a previous report, the coincidence of DS and MS showed a negative association. Here, we searched for the coincidence of DS and MS, and attempted to resolve this negative association, focusing on the gene dosage effect, by utilizing available reports.

**Case presentation:**

A 44-year-old woman with DS experienced a progressively worsening gait at onset. Auto-immuno-antibodies including aquaporin-4 antibody were negative. On the basis of brain magnetic resonance image (MRI) findings, the patient was diagnosed with possible MS. After three years, the patient demonstrated additional signs and regression symptoms. Re-examined brain MRI showed multiple new focal lesions. Based on the McDonald criteria, the patient was diagnosed with laboratory-supported defined MS.

**Discussion:**

To date, we have found only one case report in the literature describing the development of MS in a 49-year-old man with DS. The protective effect of DS against the development of MS might be mediated by a gain of function due to a gene dosage effect, and the effect of candidate antigens could be interferon alpha and beta receptors, S100B, and amyloid precursor protein (APP).

**Conclusions:**

In patients with DS, S100B and APP overexpression could protect against MS, but both correlate with the progression of Alzheimer’s neuropathological changes. S100B and APP can be seemed to be multiple pathogenesis and co-occurrence of MS with DS and Alzheimer’s dementia may advance more severely than MS without DS.

## Introduction

Down syndrome (DS) is one of the most common chromosome abnormalities with triplication of chromosome 21 (included trisomy of DS critical region) and inevitably develop Alzheimer-type neuropathology with increasing age. On the other hand, multiple sclerosis (MS) is characterized by inflammatory axonal demyelination in the central nervous system (CNS) with autoimmune dysfunction that typically results in damage to the brain, brainstem, optic nerves, and spinal cord.

DS is often associated with autoimmune diseases; however, its association with MS has rarely been reported. Here, we report the case of a 44-year-old woman with DS who progressed to MS.

## Case report

### Case presentation

A 44-year-old woman with DS presented with four-year history of intermittent claudication in the left leg and developed limb apraxia.

She was referred to our hospital by a local orthopedic surgeon following muscle weakness in the left leg. Coincidence with intermittent claudication, she developed akinetic mutism followed by progressive dementia with DS.

She was born to nonconsanguineous parents with a normal birth history and had no congenital heart disease or history of surgery. The patient had no relevant family history of MS or other diseases and the past medical history was unremarkable.

At the first visit to our hospital, when the patient was 44 years of age, we found that she had small, low-set ears, a saddle nose, and a single palmar crease, consistent with the typical findings in patients with DS. Genetic assessment, that was performed when she was 44 years of age, revealed a 47XX trisomy 21 karyotype. There was no evidence of cytomegalovirus, Epstein-Barr virus, human immunodeficiency virus, human T-lymphocyte virus, or Cryptococcus infection.

Neurological examination revealed cognitive dysfunction and left limb apraxia, and her expanded disability status scale (EDSS) score was step three.

The EDDS score is one of the most extensively accepted clinical grading scales which used to the assessment of neurological impairment and disability in MS^1)^.

On neurological examination, the function of the cranial nerves was preserved. There was mild weakness of the left lower limb. The Manual muscle test (MMT)of the left leg was three and the others were 4. Atrophy of the left quadra muscle was detected. The left patellar tendon reflex was increased and pathological reflex was positive, and the other tendon reflexes were normal and no pathological reflexes were observed.

### Brain magnetic resonance imaging (MRI) examination

Axial T2-weighted MRI and fluid-attenuated inversion recovery (FLAIR) images demonstrate multiple periventricular hyperintensities (Figure 1A and 1B). Spinal MRI revealed no suspicious areas.

**Figure 1A:**
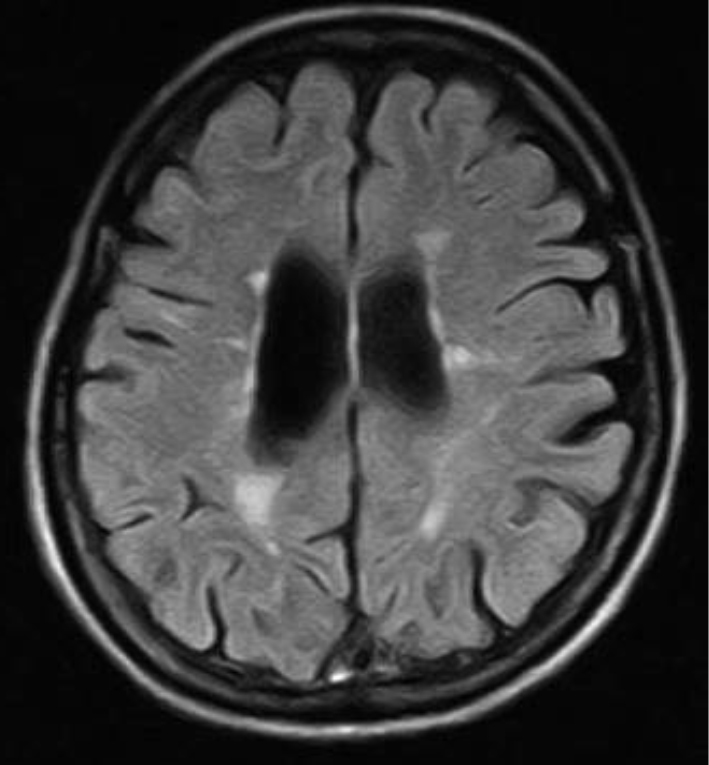
Brain MRI (fluid-attenuated inversion recovery (FLAIR)) showed multiple hyperintense white matter lesions adjacent to the bilateral ventricles at 44 years of age.

**Figure 1B:**
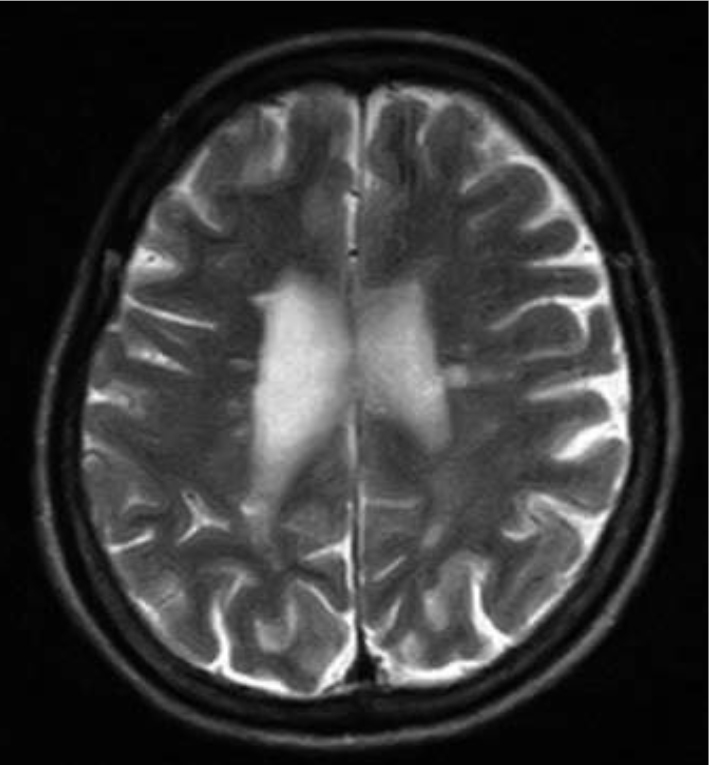
Brain MRI(T2-weighted)revealed multiple hyperintense white matter lesions adjacent to the bilateral ventricles at 44 years of age.

### Laboratory examinations

Laboratory examinations showed no abnormalities in thyroid function or Vitamin B12 or folic acid levels. The coagulation workup results (INR-PT and APTT) were within normal limits.

When considering the possibility of an autoimmune disease, we performed serial tests that showed normal C3(86 mg/dl) and C4(20.8 mg/dl) levels and were negative for antinuclear antibodies.

Antinuclear, extractable nuclear antigen, cytoplasmic, and perinuclear anti-neutrophilic cytoplasmic, anti-DNA, anti-microsomal, and auaporin-4 antibodies were negative.

Total IgG (1687 mg/dl), IgA(268 mg/dl), and IgM(140 mg/dl) levels were normal (Table 1).

Although an autoimmune disorder was initially considered after the initial assessment, her parents refused a cerebrospinal fluid (CSF) examination.

Her parents noticed a minor change in the movement left leg but did not seek CSF analysis since it did not interfere with her daily life.

The patient was diagnosed with possible multiple sclerosis (MS), and no specific treatment was administered.

**Table 1:** Laboratory examinations (The patient was 44 and 47 years of age)

anti-nuclear antibody :＜40 anti-ds DNA: negative anti-sm antibody: negative anti-SS-A/Ro antibody: negative anti-SS-B antibody: negative aquaporin-4 antibody: negative rheumatoid factor11U/ml (0-15) anti-cardiolipin antibodies＜1.2 myeloperoxidase-anti-neutrophil cytoplasmic antibody: negative proteinase 3 anti-neutrophil cytoplasmic antibody: negative Vitamin B12: 272pg/ml (180-914) (at 44 years of age) IgG 1687mg/dl (820-1740)/IgA 268mg/dl (90-400)/IgM 140mg/dl (52-270) C3: 86mg/dl (80-140) C4: 20.8mg/dl (11-34) Complement value 41U/ml (30-45) (at 44 years of age)

### A regression of symptoms after three years

During the next three years, the patient demonstrated additional signs and symptoms.

She was 47years old and could not have an ounce of strength in the left upper limb, which progressed to left hemi-weakness. She revisited our hospital for lower involuntary movement of the left upper limb, which had progressed within a few months prior to CSF evaluation. She could neither move her left upper or lower limbs nor stand alone. She also developed difficulties in standing and crawling. On revisit our hospital at 47-year-old, she needed a wheelchair with transferring help and was unable to take more than a few steps and her EDSS score was step 7.5.

Re-examined serial tests, auaporin-4 antibodies, and other tests yielded negative results. The CSF had two mononuclear cells/mm^3^, 61 mg/dl mg/dL total protein with an IgG index of 0.55, and positive oligoclonal bands.

**Figure 2:**
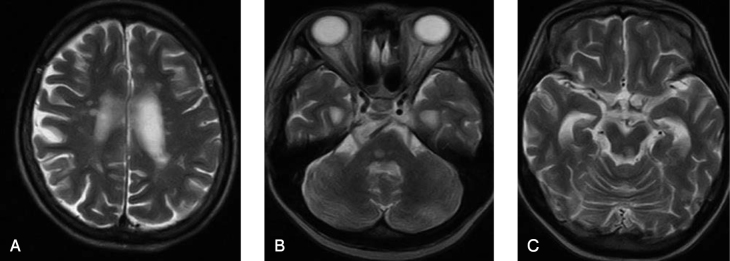
Brain MRI (T2-weighted) showed multiple newly appearing focal lesions in the subcortical area (2A), hyperintensity in the left optic nerve (2 B), and in the left midbrain tegmentium (2C) at 47 years of age.

Brain MRI showed multiple new focal lesions in the subcortical area and hyperintensity in the left optic nerve and left midbrain tegmentium (Figure 2 A.B.C). She could not communicate sufficiently with her caregivers to express complaints such as left visual impairment due to progressive dementia.

The patient had MS based on McDonald criteria, because she had two attacks, one of left limbic weakness for three years and one of left upper limb weakness for several days before revisiting our hospital, fulfilling the criteria (two or more attacks; objective clinical evidence of two or more lesions)^1)^.

She was transferred to the university hospital for specialized medical care after diagnosis without delay. Her observation period in our medical center was three years between 44 and 47 years of age. She was taken dementia into consideration and was prescribed fingolimod, the first oral therapy approved for MS at the university hospital.

## Discussion

### Multiple sclerosis

MS is an idiopathic inflammatory demyelinating disorder that occurs in approximately 70% of patients aged between 20 and 40 years^2)^. To diagnose MS, MRI findings must satisfy the criteria for the presence of lesions disseminated in space and time.

In this patient, dissemination in space refers to the evidence of demyelination in multiple distinct CNS regions, subcortical, periventricular, and left optic nerve as supratentorial lesions, and the left tegmentium of the midbrain as subtentorial lesions.

Dissemination in time correlated with the development of new demyelinating lesions in the CNS at different points in time, at 44-year-old and 47- year-old in this patient. The presence of an oligoclonal band in the CSF was used in order to determine dissemination over time^3)^.

### Co-occurrence of DS and MS

#### Autoimmune disorders in DS

When compared with the general population, people with DS have a 10-fold or even higher prevalence of certain autoimmune disorders, particularly origin-specific diseases of the endocrine glands (e.g., Hashimoto thyroiditis, Graves’ disease, and type I diabetes mellitus), as well as of the gut (celiac disease) and skin (alopecia areata and vitiligo) and the onset of the aforementioned autoimmune disorders in patients with DS is typically lower than that in the general population^4, 5)^.

Weilbach et al. reviewed only one case of DS associated with MS published to date and proposed that there is a negative association of DS and MS^4)^. To date, we found only one case report in the literature describing the development of MS in a 49- year-old man with DS who experienced paroxysmal pelvic pain at onset. Although concomitant DS and MS were not the main focus of the study, the authors identified an effective combination of antiepileptic drugs for pelvic pain^6)^.

The protective effect of DS against MS might be mediated by a gain-of-function due to a gene dosage effect. Candidate genes for protection against MS on chromosome 21 seem to be interferon alpha and beta receptor (IFNAR)^4)^, S100B^4)^, and amyloid precursor protein (APP)^7)^.

#### Interferon alpha and beta receptors

Interferon dysregulation in individuals with DS is well established and produced by trisomy of chromosome 21 which contains a cluster of four out of six interferon receptor genes: IFNAR1, IFNAR2, IFNGR2 and IL10RB^8)^.

These genes have been shown to have variable expression in multiple cell types in individuals with DS and can account for baseline interferon activity, as would be seen in an individual having increased interferon signature^8)^.

Type I interferons include interferon α, interferon β, interferon ε, interferon κ and interferon ω in humans, as well as interferon δ signaling via the type I receptor (IFNAR) composed of the receptor subunits IFNAR1 and IFNAR2.

Malle et al. proposed that Type I interferonopathies was caused by type I interferon overproduction or hyperresponsiveness to type I interferon and DS could be considered to belong to the one of the type I interferonopathies^9)^.

Central nervous system involvement in interferonopathies manifests as developmental delay, seizures and these manifestations all occur in DS^9)^.

Type 1 interferons are of particular importance in MS because interferon-beta in humans appears to be protective against autoimmunity associated with MS, and interferon-1b was the first disease- modifying agent for the treatment of relapsing-remitting MS^4)^.

The constant activation of interferon signaling may explain many aspects of DS, including neurological problems and protection against MS.

#### Amyloid precursor proteins in DS

In adults with DS, neuronal loss, neurofibrillary and neuritic Aβ plaque pathologies are consistent with Alzheimer disease. These pathological changes are thought to underlie the neuropsychological and physiological changes in older individuals with DS. Two chromosome 21-based gene products, amyloid precursor protein (APP) and S100B, have been implicated in these neuronal and interstitial changes, and in protection against MS.

The overproduction through the gene dose effect of APP localized at 21q21.2-3, near the critical region for DS, leads to early onset Aβ plaques^10)^.

APP synthesized within the neuronal perikarya (gray matter) is bound to the membranes of secretory granules and transported along the axons (white matter). During demyelination, APP is produced by astrocytes, upregulated, and distributed along the injured axons. Patients with MS present with higher levels of APP than controls, and axons that are positive for APP in patients with MS show a correlation with the spread of the lesion^7)^.

Based on the assumption that APP is a growth factor and a mediator of cell adhesion, and overexpression of APP through a gene dosage effect, APP could be a candidate gene against MS.

#### S100 protein in DS

S100B, a small, soluble, astrocyte-derived homodimeric cytokine encoded by a gene mapped to the DS-critical region on chromosome21, exerts trophic effects on neurons and glia, including proliferation and differentiation. Neuritogenic and survival-promoting actions of S100B are associated with intracellular calcium modulation and development-related events^11)^. This S100B overexpression increases progressively throughout life in DS^12)^ and, in post- adolescent DS patients. The degree of this S100B overexpression correlates with the degree of Aβ deposition in brain^13)^, suggesting a continuing role for S100B overexpression in either the maintenance of or promotion of progression of Alzheimer neuropathological changes.

On the assumption of that S100B functions include neuronal proliferation, synaptogenesis and dendritic development and differentiating oligodendrocytes, over-expression of S100B is important for protect MS and one of the candidate antigen for against MS.

Camponeschi et al. demonstrated that the inhibitor of astrocytic S100B synthesis with arundic acid in the MS animal model, a significant reduction of astrogliosis, demyelination, proinflammatory cytokine expression were recognized^14)^. On the assumption of that the overexpression of S100B through its receptor is regarded to act via the activation of nuclear factor (NF)-κB proinflammatory cascade^14)^, the inhibitor of astrocytic S100B synthesis with arundic acid lead to decrease the degree of inflammatory process. Nevertheless the biomolecular evaluation of S100B in brain tissue showed that the protein was not reduced as compared to in the control animals^14)^.

They proposed the targeting the S100B protein to be considered for MS treatment^14)^.

In patients with DS, S100B and APP overexpression could protect against MS, but both correlate with the promotion of the progression of Alzheimer’s neuropathological changes.

S100B and APP can be seemed to be multiple pathogenesis.

#### Once DS get MS

In a previous report of patients with MS who developed Alzheimer’s disease (AD), there was significantly more acute axonal injury in the cortex and white matter of inactive MS patients with AD than in pathologically inactive MS patients without AD^15)^. The EDSS in this patient with no therapy progressed from step three to 7.5 within only three years. A cohort study of long-term disability trajectories reported by Lozak et al. showed that approximately 90 percent of patients with EDDS step three with no therapy were retained within EDDS Step 6 over three years^16)^. Based on the prognosis of the cohort study, the disability progression of MS with DS and Alzheimer’s dementia (pathological changes) may advance more severely than without DS.

## Sumnary

1. Here, we report the case of a 44-year-old female with DS who progressed to MS. The co-occurrence of DS and MS occurs less frequently, as previously reported, than expected based on random association.

2. The protective effect of DS against the development of MS might be mediated by a gain of function due to a gene dosage effect, and the effect of candidate antigens could be interferon alpha and beta receptors, S100, and APP.

3. Coexistence of MS with DS may progress more severely than MS without DS.

## Funding

No funding was received.

## Author contributions

All authors read and approved the final manuscript. YA is the main author of the manuscript. CM made substantial contributions to data collection. All authors meet the ICMJE authorship criteria.

## Informed consent

Informed consent by oral explanation for publication of this article was obtained from the patient’s guardian.

## Conflicts of interest statement

The content of this article is solely the responsibility of the authors. The authors declare that there are no conflicts of interest.
